# Efficacy and safety of PT20, an iron-based phosphate binder, for the treatment of hyperphosphataemia: a randomized, double-blind, placebo-controlled, dose-ranging, Phase IIb study in patients with haemodialysis-dependent chronic kidney disease

**DOI:** 10.1093/ndt/gfaa116

**Published:** 2020-07-11

**Authors:** Mark Sampson, Nuno Faria, Jonathan J Powell, the PEACH study investigators

**Affiliations:** 1 Shield Therapeutics PLC, London, UK; 2 Department of Veterinary Medicine, University of Cambridge, Cambridge, UK; 3 Medical Research Council Elsie Widdowson Laboratory, Cambridge, UK

**Keywords:** chronic kidney disease, ferric oxide, hyperphosphataemia, phosphate binder, PT20

## Abstract

**Background:**

Hyperphosphataemia is a common complication of chronic kidney disease (CKD). PT20 (ferric iron oxide adipate) is an investigational molecule engineered to offer enhanced phosphate-binding properties relative to other phosphate binders.

**Methods:**

In this double-blind, parallel-group, placebo-controlled, dose-ranging study (ClinicalTrials.gov identifier NCT02151643), the efficacy and safety of 28 days of oral PT20 treatment were evaluated in patients with dialysis-dependent CKD. Participants were randomly assigned in an 8:8:8:13:13 ratio to receive PT20 (400, 800, 1600 or 3200 mg) or placebo three times daily.

**Results:**

Among 153 participants, 129 completed treatment [7 discontinued because of adverse events (AEs), 2 because of hyperphosphataemia and 15 for other reasons]. PT20 treatment for 28 days resulted in a statistically significant and dose-dependent reduction in serum phosphate concentration. There were no statistically significant effects of PT20 treatment on changes in haemoglobin or ferritin concentrations or transferrin saturation between Days 1 and 29. The incidence of treatment-emergent AEs was broadly similar across the PT20 and placebo groups (42–59% versus 44%). The most common PT20 treatment-related AEs were gastrointestinal, primarily diarrhoea (13–18%) and discoloured faeces (3–23%). No serious AEs were considered to be related to study treatment. There were no clinically significant changes in laboratory results reflecting acid/base status or increases in ferritin that could indicate the absorption of components of PT20.

**Conclusions:**

In this first study investigating the efficacy and safety of PT20 in patients with hyperphosphataemia and dialysis-dependent CKD, PT20 significantly lowered serum phosphate concentrations and was generally well tolerated.

## Graphical Abstract

**Figure:**
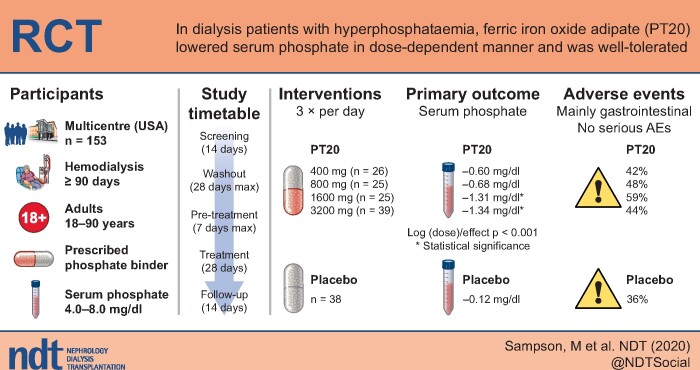

## INTRODUCTION

Elevated serum phosphate is a common complication of chronic kidney disease (CKD) and is associated with hyperparathyroidism, metastatic calcification and cardiovascular morbidity and mortality in patients requiring haemodialysis [1, 2]. In a large international practice study, approximately half of dialysis patients had serum phosphate concentrations above the recommended target of 5.5 mg/dL [3, 4].

Strategies to control hyperphosphataemia include dietary phosphate restriction, dialysis intensification and use of oral phosphate binders that sequester phosphate in the small intestine, preventing absorption [5]. Of the available phosphate-binding drugs, conventional calcium-based compounds (calcium acetate and calcium carbonate) are efficacious but have been associated with adverse events (AEs), including hypercalcaemia, adynamic bone disease, progressive vascular calcification and increased mortality [6, 7]. Among the more recently developed calcium-free phosphate binders, sevelamer carbonate can cause significant gastrointestinal (GI) AEs, which, along with a high pill burden and negative effect on other medication, may limit compliance [6–8]. Lanthanum carbonate may accumulate in bone, with the potential for long-term systemic toxicity [6–9].

Ferric compounds can sequester dietary phosphate [10] and two (sucroferric oxyhydroxide and ferric citrate) are now approved for the treatment of hyperphosphataemia in patients requiring dialysis [11–13]. These molecules have shown similar efficacy to sevelamer in controlling phosphate levels, with reduced pill burden, although they have been associated with potentially treatment-limiting GI events such as diarrhoea (particularly in the first 6 months of treatment) and, with long-term treatment, hypophosphataemia and iron accumulation [14–17]. Non-adherence remains a challenge to the efficacy of all currently available phosphate binders [18].

PT20 is an investigational combination of ferric iron oxide and the dietary ligand adipic acid. It was specifically engineered for enhanced phosphate-binding capacity and affinity, thereby potentially reducing the pill burden needed to control phosphate levels or leading to lower phosphate levels with the same pill burden as other available phosphate binders [8, 19, 20]. Interstitial mineral hydroxide chemistry was used to incorporate adipic acid into the primary structure of the ferric iron oxide, increasing the available surface area for phosphate binding. As relatively weak ligands, adipate moieties are substituted by phosphate more easily than the surface hydroxyl groups of unmodified ferric iron oxide, resulting in a 2- to 3-fold higher *in vitro* phosphate-binding efficiency than unmodified ferric iron oxide [19]. Both components are well characterized, with ferric iron oxide used as an oral iron supplement and in medicinal products and adipic acid being an organic acid found in beets and sugarcane that is commonly used as a food additive and classified as ‘generally recognized as safe’ [21–23].

The objectives of this trial were to evaluate the efficacy and safety of 28 days of oral PT20 administration at a range of doses, taking into account the need for dose titration based on diet and extent of disease, in patients with dialysis-dependent CKD.

## MATERIALS AND METHODS

### Study design and setting

This Phase 2b, randomized, double-blind, parallel-group, placebo-controlled, dose-ranging study was conducted in 21 nephrology centres in the USA between May 2014 and March 2015 (ClinicalTrials.gov identifier NCT02151643). The study comprised five periods: screening (14 days maximum, after which current phosphate binders were stopped), washout (28 days maximum), pre-treatment (7 days maximum), treatment (28 days) and follow-up (14 days). After screening, eligible patients were randomly assigned in an 8:8:8:13:13 ratio to receive PT20 400 mg (1.2 g/day), 800 mg (2.4 g/day), 1600 mg (4.8 g/day) or 3200 mg (9.6 g/day) or placebo. Treatment was given three times daily (TID) with food. Randomization was stratified according to patients’ pre-randomization serum phosphate levels (<7.5 or ≥7.5 mg/dL). The randomization list was generated by an independent statistical provider and randomization was performed centrally using an interactive web response system.

The study was approved by the appropriate institutional review boards and conducted in accordance with the Declaration of Helsinki and International Council for Harmonisation of Technical Requirements for Pharmaceuticals for Human Use guidelines on good clinical practice. All patients provided written informed consent.

### Patients

Adults (ages 18–90 years) with CKD were eligible for inclusion if, before screening, they had received maintenance haemodialysis three times weekly for ≥90 days, had been prescribed at least one oral phosphate binder and had a serum phosphate concentration of 4.0–8.0 mg/dL for ≥28 days. Patients needed an increase in phosphate of ≥1.0 mg/dL and a minimum phosphate level of 5.5 mg/dL to continue past the washout period.

At screening, patients were required to have the following: serum ferritin <1000 ng/mL (ferritin 700–999 ng/mL required consent of the safety monitoring committee), pre-dialysis serum bicarbonate ≥18 mg/dL, intact parathyroid hormone <1000 pg/mL and liver function measures within twice the upper limit of central laboratory values. A urea reduction ratio ≥65% or a *K_t_/V* ≥1.3 as the most recent value during 28 days before washout was also required, along with stable doses of calcimimetics or vitamin D for 14 days before washout to start of study treatment.

The main exclusion criteria were as follows: GI disorder or surgery that could impair absorption or tolerance of oral iron; inflammatory bowel disease; contraindication of iron preparations; severe chronic lung disease or carbon dioxide retention; history of immunodeficiency diseases or active hepatitis B or C; current or recent treatment for malignancy; pregnancy, lactation or childbearing potential without adequate contraception and recent or planned hospitalization (except for vascular access procedures).

Patients taking oral iron supplements discontinued these at the beginning of the washout period. Intravenous (IV) iron could be used after the first 2 weeks of study treatment if clinically necessary and if transferrin saturation was <30% and ferritin was <500 ng/mL before each administration. Multivitamin or mineral supplements were allowed if daily iron intake was ≤30 mg. Calcium supplements had to be taken ≥2 h before or after food. Doses of niacin or niacin/nicotinamide had to be stable from 14 days before screening to the end of study treatment. Calcium could not be used as an additional phosphate binder.

### Study treatment

Study medication was supplied as PT20 (ferric iron oxide adipate) 400 and 800 mg tablets or placebo tablets matched for colour, weight and composition of non-active ingredients to maintain double-blind conditions. Study treatment was taken orally TID (with breakfast/snack, lunch/snack and evening meal/snack) over 28 days. Patients were requested to follow a standard recommended diet. Study treatment was discontinued if serum bicarbonate was <15 mmol/L or serum phosphate was ≥9.5 or <3.0 mg/dL at two consecutive visits during the treatment period or if serum ferritin was >1200 ng/mL at any time. No rescue treatment was permitted as part of the trial.

### Outcome measures

The primary efficacy endpoint was a change in serum phosphate concentration from Days 1 to 29. Secondary efficacy endpoints were changes in haemoglobin and ferritin concentrations and transferrin saturation from Days 1 to 29 (all assessed on Days 1, 8, 15 and 29).

Safety outcomes included treatment-emergent adverse events (TEAEs), GI AEs assessed according to the GI symptom rating scale (GSRS) [24] and clinically significant laboratory abnormalities. The GSRS is a relatively brief questionnaire covering the main GI symptoms that may be associated with the use of non-calcium phosphate binders in patients undergoing haemodialysis. It includes 15 items divided into five subscales (diarrhoea, indigestion, abdominal pain, constipation and reflux), which are assessed on a 7-point Likert scale (1 = no discomfort at all, 7 = very severe discomfort), with higher scores indicating a greater symptom impact [25]. An independent safety reviewer assessed unblinded data on iron measures and related AEs. Compliance was assessed by evaluating the proportion of dispensed tablets that were returned.

The US Food and Drug Administration requested that a pharmacokinetic substudy be added to the study protocol: those results will be reported separately.

### Statistical analyses

We planned the randomization ratio of 8:8:8:13:13 (low to high PT20 dose, placebo) to give >90% power to detect either a statistically significant slope for the dose–response relationship or, if the relationship is not linear, a statistically significant difference between the highest-dose group and the placebo group, using a two-sided 0.05 significance level. We assumed a common standard deviation (SD) of 1.844 mg/dL, estimated as the upper 95% confidence limit for the pooled estimate from two similar dose-ranging studies [26, 27]. All data analyses were conducted using SAS version 9.3 (SAS Institute, Cary, NC, USA).

The primary analysis was conducted in the intention-to-treat (ITT) population (all randomized patients) using a two-step process. First, a linear model on the log(dose) scale (with placebo equally spaced below the lowest PT20 dose), stratified by pre-randomization serum phosphate concentration (<7.5 or ≥7.5 mg/dL) was fitted to the data. If linear model lack of fit was not found (*F*-test at 0.05 level), a slope significantly different from zero was an indication of a significant dose response. Missing values were imputed using the last observation carried forward (LOCF) methodology. These dose–response analyses were also performed in the modified ITT (mITT) population (all randomized patients who received at least one treatment dose).

Sensitivity analyses were performed on the ITT population using two imputation methods for missing Day 29 values: baseline observation carried forward (i.e. zero change from baseline assumed) and multiple imputation (MI; SAS MI procedure) with the variables baseline serum phosphate and treatment group to generate 20 datasets. Linear model dose–response analysis was conducted on each dataset and MI analysis results were combined using SAS MIANALYSE. The primary analysis was also repeated in the per-protocol (PP) population, defined as all randomized patients who were understood to be without major protocol deviations at the time of the database lock, using the LOCF methodology.

The change in serum phosphate concentration was assessed using a mixed model repeated measures (MMRM) analysis, including fixed categorical effects of treatment, week and treatment × week interaction, and continuous fixed covariates of baseline serum phosphate and baseline serum phosphate × week interaction (ITT).

Changes in haemoglobin, ferritin and transferrin saturation were analysed using analysis of covariance (ANCOVA) for patients with Day 1 and Day 29 values. Since 24 participants received IV iron during the study, a *post hoc* analysis deleting these individuals from the ITT population to test for effects on iron measures was planned before database lock.

AEs and other safety assessments were conducted in the safety population, comprising all patients who received at least one dose of study drug and who had at least one subsequent contact with the investigator.

## RESULTS

### Patient characteristics

**FIGURE 1:**
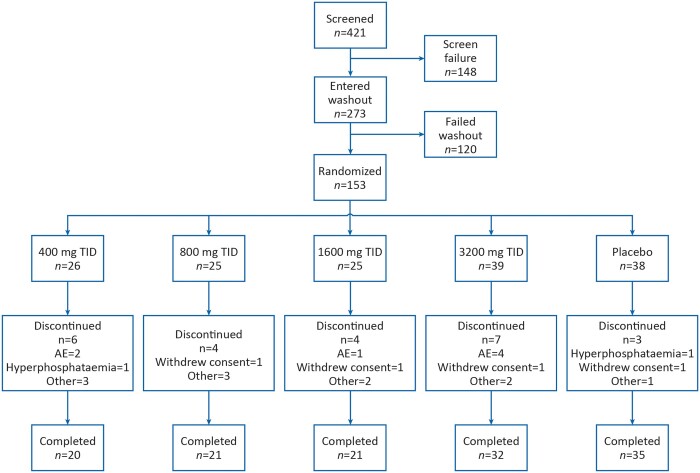
Patient disposition.

Of 421 patients screened, 153 met criteria for eligibility and continuation after washout and were randomly assigned to receive PT20 400 mg (*n *=* *26), 800 mg (*n *=* *25*)*, 1600 mg (*n = *25), 3200 mg (*n *=* *39) or placebo (*n *=* *38) (Figure 1). Twelve randomized patients did not receive the study drug (the mITT and safety populations therefore comprised 141 patients) and 36 patients had major protocol deviations at the time of database lock (the PP population therefore comprised 117 patients). Most of these deviations involved the use of prohibited iron (*n *=* *23).

The mean ± SD treatment compliance was 75.4 ± 16.6% for PT20 400 mg, 68.1 ± 21.1% for 800 mg, 75.8 ± 19.3% for 1600 mg and 70.9 ± 25.1% for 3200 mg; the mean compliance in the placebo group was 78.9 ± 17.0%. Fourteen patients had low compliance (<50%): 8 of these discontinued because of an AE or for other reasons and 2 withdrew consent voluntarily; 12 were included in the PP population.

Overall, 59% of the patients were men and 54% were Caucasian. Their median age was 57 years. Patient demographics and baseline characteristics appeared generally similar across groups (statistical analysis not done) (Table 1). The most common pre-existing medical conditions were in the endocrine/metabolic and cardiovascular systems, reflective of a population with CKD.

**Table 1:** Patient demographics and baseline characteristics (ITT population)

Characteristics	Placebo (TID)	PT20 (TID)			
	(*n *=* *38)	400* *mg (*n *=* *26)	800* *mg (*n *=* *25)	1600* *mg (*n *=* *25)	3200* *mg (*n *=* *39)
Age (years), mean ± SD	54.9 ± 12.5	60.5 ± 12.2	57.7 ± 12.5	60.0 ± 12.5	55.9 ± 11.6
Men, *n* (%)	23 (61)	18 (69)	15 (60)	13 (52)	22 (56)
Hispanic/Latino, *n* (%)	15 (39)	10 (38)	10 (40)	9 (36)	16 (41)
Race, *n* (%)					
Caucasian	18 (47)	14 (54)	12 (48)	15 (60)	23 (59)
Black or African American	16 (42)	7 (27)	9 (36)	9 (36)	12 (31)
Other^a^	4 (11)	5 (19)	4 (16)	1 (4)	4 (10)
Medical history,^b^*n* (%)					
Endocrine/metabolic	38 (100)	26 (100)	24 (96)	25 (100)	39 (100)
Cardiovascular	37 (97)	26 (100)	24 (96)	24 (96)	38 (97)
Genitourinary/reproductive	25 (66)	19 (73)	20 (80)	19 (76)	28 (72)

a African Indian or native Alaskan, Asian, native Hawaiian or other Pacific Islander.

b Body systems with the highest incidence of pre-existing medical conditions.

### Effect of PT20 on serum phosphate

**FIGURE 2:**
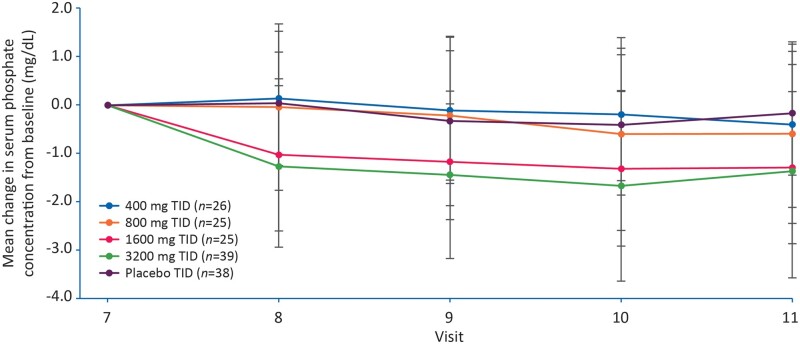
Mean change in serum phosphate concentration from Day 1 to 29 in patients receiving placebo or PT20 400, 800, 1600 or 3200 mg, each given TID (ITT population).

**FIGURE 3:**
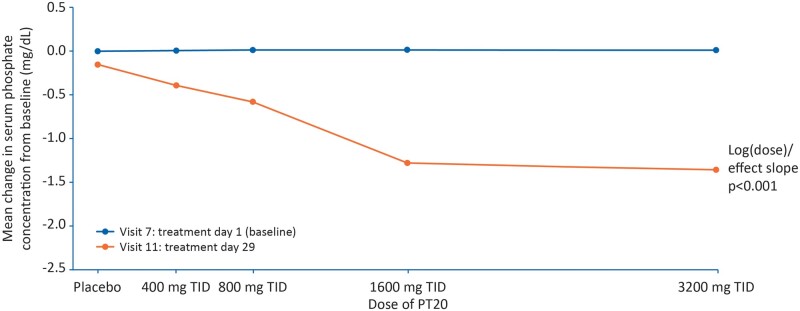
Mean change in serum phosphate concentration from Day 1 to 29, by dose group (ITT population). The blue data points represent baseline values for the placebo group and each of the treatment groups in ascending order. The red data points show the change from baseline assessed at Day 29 for the placebo group and each of the treatment groups in ascending order. A significant dose-dependent change in serum phosphate concentration over time is shown by the log(dose)/effect slope (P < 0.001).

Serum phosphate concentrations were similar across all groups at baseline and decreased from Day 1 to 29 in all groups (Table 2 and Figure 2). The magnitude of the reductions in serum phosphate levels increased linearly with increasing PT20 dose, being most notable in the 1600 and 3200 mg dose groups (Figure 3). The P-value for the log(dose)/effect slope in Figure 3 was <0.001, demonstrating that the dose-dependent change in serum phosphate concentration over time was statistically significant. These findings were reflected in the mITT and PP populations (P < 0.001 for ITT and mITT, P = 0.006 for PP). The P-values for linear model lack of fit were 0.784 (ITT population), 0.743 (mITT) and 0.914 (PP).

**Table 2:** Change in serum phosphate concentration over time (ITT population)

Serum phosphate (mg/dL)	Placebo (TID)		PT20 (TID)														
			400* *mg			800* *mg				1600* *mg				3200* *mg			
	*n*	Mean ± SD	*N*	Mean ± SD		*n*		Mean ± SD		*n*		Mean ± SD		*n*	Mean ± SD		
Treatment period																	
Day 1 (baseline)	37	7.01 ± 1.43	25	6.90 ± 1.68		22		7.06 ± 1.92		23		7.57 ± 1.33		39		7.13 ± 1.94	
Day 8	36	7.11 ± 1.18	22	7.13 ± 1.82		19		7.11 ± 2.03		23		6.55 ± 1.41		37		5.80 ± 1.55	
Day 15	37	6.68 ± 1.80	24	6.84 ± 1.98		21		6.93 ± 1.62		22		6.31 ± 1.37		34		5.59 ± 1.29	
Day 22	35	6.67 ± 1.63	21	6.33 ± 1.57		20		6.59 ± 1.62		22		6.16 ± 1.51		30		5.50 ± 1.74	
Day 29	35	6.89 ± 1.30	20	6.30 ± 1.92		20		6.38 ± 1.56		20		6.26 ± 1.22		32		5.79 ± 1.59	
Follow-up^a^																	
Day 36	35	6.05 ± 1.34	20	5.87 ± 1.66		20		6.19 ± 1.10		21		5.99 ± 1.50		32		5.49 ± 1.38	
Day 43	16	6.04 ± 1.20	10	6.38 ± 1.29		12		5.68 ± 1.03		6		5.28 ± 1.86		27		5.45 ± 1.65	

a Where possible, patients who had at least one dose of study medication and withdrew early (excluding those who withdrew consent) provided a blood sample on Day 29 and on either Day 36 or 43.

The results of the sensitivity analyses were consistent with the findings above (data not shown). The MMRM analysis showed a statistically significant effect for treatment group (P < 0.001) and baseline serum phosphate concentration (P < 0.001). To further explore the dose effect, each PT20 dose group was compared with placebo: statistically significant effects were found for the 1600 and 3200 mg groups, with estimated mean treatment differences of –0.705 [standard error (SE) 0.289] and –1.195 (SE 0.255), respectively (one-sided upper 97.5% confidence interval –0.050 and –0.618, respectively).

### Effect of PT20 on haematological measures

In the ITT population, mean haemoglobin and ferritin concentrations and transferrin saturation were broadly similar across treatment groups at Day 1. There was no statistically significant effect of treatment group on haemoglobin or ferritin concentrations or on transferrin saturation between Day 1 and 29 (ANCOVA P > 0.05 for each measure, ITT population).

Calcium × phosphate levels were broadly similar across treatment groups at Day 1; there was a significant treatment effect with respect to the change in these levels [mean ANCOVA estimate for the change from baseline relative to placebo: –2.502 (SE 3.331) for 400 mg, 0.272 (SE 3.388) for 800 mg, –6.005 (SE 3.348) for 1600 mg and –10.371 (SE 2.935) for 3200 mg], with the 1600 and 3200 mg dose groups having significantly larger changes than the placebo group (P = 0.004).

When data for 24 patients who received IV iron during the study were removed in a *post hoc* analysis (Table 3), the results were similar to those above: no statistically significant between-group changes were found for haemoglobin, serum ferritin or transferrin saturation, whereas for calcium × phosphate, the active treatment groups had a statistically significant greater change from baseline than the placebo group [mean ANCOVA estimate for the change from baseline relative to placebo: −6.618 (SE 3.540) for 400 mg, –0.404 (SE 3.617) for 800 mg, –5.968 (SE 3.639) for 1600 mg and −9.574 (SE 3.136) for 3200 mg; P < 0.02].

**Table 3:** Change in haematological measures over time (ITT population excluding patients who used intravenous iron; *post hoc* analysis)

Measures	Placebo (TID)		PT20 (TID)							
			400* *mg		800* *mg		1600* *mg		3200* *mg	
	*n*	Mean ± SD	*n*	Mean ± SD	*n*	Mean ± SD	*n*	Mean ± SD	*n*	Mean ± SD
Haemoglobin (g/dL)										
Day 1 (baseline)	28	10.73 ± 1.45	21	10.75 ± 0.82	18	10.72 ± 0.97	19	10.92 ± 1.13	32	10.65 ± 1.08
Day 29	25	10.52 ± 1.41	17	10.79 ± 1.31	17	10.46 ± 0.84	16	10.52 ± 0.91	26	10.85 ± 1.01
Change from Day 1 to 29	22	−0.14 ± 0.93	16	−0.05 ± 0.89	17	−0.35 ± 1.11	16	−0.27 ± 1.03	26	0.15 ± 0.92
Change from Day 1 to 29, median (range)	22	−0.25 (−1.4–2.5)	16	0.30 (−1.7–1.1)	17	−0.10 (−3.0–1.2)	16	−0.05 (−2.9–1.3)	26	0.05 (−1.4–2.4)
Serum ferritin (ng/mL)										
Day 1 (baseline)	30	593.5 ± 273.9	22	587.0 ± 237.1	18	480.6 ± 168.8	19	511.5 ± 273.1	33	537.6 ± 185.3
Day 29	28	522.5 ± 223.8	17	609.4 ± 284.9	17	538.8 ± 306.6	16	545.3 ± 251.6	26	556.3 ± 192.0
Change from Day 1 to 29	27	−82.4 ± 151.1	17	1.4 ± 136.4	17	47.7 ± 243.7	16	5.3 ± 116.7	26	39.8 ± 145.5
Change from Day 1 to 29, median (range)	27	−90.0 (−468–299)	17	−53.0 (−159–264)	17	−29.0 (−256–630)	16	−16.5 (−120–368)	26	18.0 (−190–451)
Transferrin saturation (%)										
Day 1 (baseline)	29	30.9 ± 8.9	22	30.1 ± 10.1	18	32.2 ± 16.2	18	33.6 ± 11.8	31	37.7 ± 14.8
Day 29	28	29.6 ± 9.8	17	32.9 ± 8.8	17	32.9 ± 12.6	16	36.7 ± 11.6	26	36.2 ± 11.2
Change from Day 1 to 29	26	−1.31 ± 8.81	17	0.82 ± 8.30	17	–0.12 ± 12.52	15	1.53 ± 6.94	25	–5.16 ± 15.71
Change from Day 1 to 29, median (range)	26	−1.50 (−20.0–22.0)	17	−1.00 (−11.0–19.0)	17	−1.00 (−28.0–26.0)	15	2.00 (−10.0–16.0)	25	−4.0 (−38.0–30.0)

### Safety

A total of 157 TEAEs were reported in 66 patients (47%). The incidence of TEAEs appeared broadly similar across the PT20 and placebo groups, although the trial was not designed for statistical comparisons of safety data across groups (Table 4). The most common TEAEs were diarrhoea, discoloured faeces, constipation, dyspnoea, nausea, upper respiratory tract infection and vomiting. Most TEAEs were mild [93/157 (59%)] or moderate [49/157 (31.2%)] and the TEAE profile by maximum intensity appeared to be similar between the PT20 and placebo groups.

**Table 4:** Incidence of TEAEs (safety population)

TEAEs	Placebo (TID)	PT20 (TID)				Total
	(*n *=* *36)	400* *mg (*n *=* *24)	800* *mg (*n *=* *23)	1600* *mg (*n *=* *22)	3200* *mg (*n *=* *36)	(*N *=* *141)
Any TEAE, *n* (%)	16 (44)	10 (42)	11 (48)	13 (59)	16 (44)	66 (47)
Any discontinuations due to TEAEs, *n* (%)	0	1 (4)	1 (4)	2 (9)	3 (8)	7 (5)
Any serious TEAEs^a^, *n* (%)	5 (14)	0	1 (4)	1 (5)	5 (14)	12 (9)
Any treatment-related TEAEs, *n* (%)	2 (6)	4 (17)	5 (22)	5 (23)	8 (22)	24 (17)
TEAEs in >5% of patients in any treatment group, *n* (%)						
Diarrhoea	2 (6)	3 (13)	3 (13)	4 (18)	6 (17)	18 (13)
Discoloured faeces	0	2 (8)	3 (13)	5 (23)	1 (3)	11 (8)
Constipation	4 (11)	0	0	0	1 (3)	5 (4)
Dyspnoea	1 (3)	2 (8)	0	0	1 (3)	4 (3)
Nausea	0	2 (8)	1 (4)	0	1 (3)	4 (3)
Upper respiratory tract infection	0	0	0	2 (9)	2 (6)	4 (3)
Vomiting	0	0	1 (4)	0	3 (8)	4 (3)
Cough	2 (6)	0	1 (4)	0	0	3 (2)
Upper abdominal pain	0	1 (4)	0	2 (9)	0	3 (2)
Urinary tract infection	1 (3)	0	0	0	2 (6)	3 (2)
Abdominal distension	0	0	0	0	2 (6)	2 (1)
Gastro-oesophageal reflux disease	0	0	0	0	2 (6)	2 (1)
Pain in extremity	2 (6)	0	0	0	0	2 (1)
Toothache	0	0	2 (9)	0	0	2 (1)

a One case each of neck pain (800 mg group); road traffic accident (1600 mg group); bacterial arthritis, osteomyelitis, pneumonia, urinary tract infection, catheter site pain and dyspnoea (all 3200 mg group) and staphylococcal bacteraemia, acute coronary syndrome, lower GI haemorrhage, hypervolaemia and femoral artery aneurysm (all placebo group). One patient could have more than one serious TEAE.

Eleven TEAEs in seven PT20 recipients (5%) led to early discontinuation from the study: one patient (4%) each in the 400 and 800 mg groups, two patients (9%) in the 1600 mg group and three patients (8%) in the 3200 mg group. No patients discontinued early from the placebo group. TEAEs leading to early discontinuation were diarrhoea and discoloured faeces (each in two patients), abdominal discomfort, vomiting, dysphagia, flatulence, vertigo, chest pain and nausea (each in one patient), with some patients experiencing more than one event. Eight TEAEs in six patients (4%) led to treatment interruption, one patient each in the 400 mg and placebo groups and two patients each in the 1600 and 3200 mg groups.

Overall, 44 TEAEs in 24 patients (17%) were considered to be related to study medication. The incidence of treatment-related TEAEs was similar across all PT20 dose groups (17–23%) and higher than in the placebo group (6%) (Table 4). Diarrhoea and discoloured faeces were the most commonly reported treatment-related TEAEs.

Thirteen serious TEAEs were reported in 12 patients; none was considered to be related to study treatment (Table 4). There were no deaths in the randomized population, but one patient died before randomization.

There were no clinically significant changes in haematology or coagulation measures. There were no apparent differences between the PT20 and placebo groups with respect to changes in clinical chemistry values, liver enzymes, vital signs and electrocardiograms and no clinically significant differences with respect to iron tests.

In general, GSRS total scores did not differ significantly in the five groups on Days 1, 15 and 29 (Table 5). On Day 1, the GSRS score was significantly higher in the 400 mg group than in the placebo group (P = 0.037). Similarly, changes from Day 1 were generally not statistically significant except in the 1600 mg group on Day 15 (P = 0.049) and in the 800 mg group on Day 29 (P = 0.047).

**Table 5:** Change in overall score on the GSRS (safety population)

GSRS	Placebo (TID)		PT20 (TID)							
			400 mg		800 mg		1600 mg		3200 mg	
	*n*	Mean ± SD	*n*	Mean ± SD	*n*	Mean ± SD	*n*	Mean ± SD	*n*	Mean ± SD
Day 1 (baseline)	36	7.0 ± 7.98	24	7.8 ± 6.83	22	6.3 ± 12.44	22	6.5 ± 5.80	36	6.1 ± 7.07
Day 15	35	5.6 ± 6.21	24	10.3 ± 9.49	21	5.6 ± 8.85	20	5.0 ± 7.05	35	8.7 ± 10.27
Change from Day 1 to 15	35	−1.4 ± 6.21	24	2.5 ± 9.28	21	−0.7 ± 6.22	20	−2.2 ± 7.11	35	2.8 ± 8.13
Day 29	34	7.1 ± 8.49	22	7.5 ± 6.66	21	9.0 ± 15.42	19	7.6 ± 10.85	31	8.0 ± 12.28
Change from Day 1 to 29	34	0.1 ± 5.16	22	−0.9 ± 3.75	21	2.8 ± 5.97	19	0.4 ± 9.80	31	1.6 ± 8.48

## DISCUSSION

In this first study of PT20 in the CKD population, treatment for 28 days resulted in a statistically significant and dose-dependent reduction in serum phosphate, compared with placebo, in dialysis-dependent patients with hyperphosphataemia. This reduction was demonstrated in ITT, mITT and PP populations and using three different methods of imputation, highlighting the robustness of the data.

Our data show that PT20 at 4.8 g/day reduces serum phosphate by 1.3 mg/dL after 28 days. Such a reduction in serum phosphate with a relatively low dose may be advantageous, given that several available phosphate binders are inadequate for maintaining normal phosphate levels in patients on dialysis even when dietary phosphate intake is restricted (which is increasingly difficult with the widespread use of phosphate-containing food additives) [28]. Indeed, the most recently introduced phosphate binders, sevelamer (∼7 g/day) and lanthanum (2.7 g/day), have been shown to reduce serum phosphorus by ∼0.2–0.3 mg/dL or 200–250 mg/day [28, 29]. The latter value is below the reduction of 300–400 mg/day estimated to be necessary to maintain serum phosphate levels at near-normal levels in patients undergoing dialysis [30].

PT20 was generally well tolerated. Fewer than 7% of PT20-treated patients withdrew because of TEAEs and no serious AEs were considered to be related to treatment. AEs were mainly GI in nature, generally of mild intensity and not related to dose. The GSRS questionnaire results did not show any increased GI symptoms that could be clearly attributed to study treatment. As with sucroferric oxyhydroxide [14], the GI AEs most commonly occurring with PT20 were diarrhoea and discoloured faeces; only one patient (in the 3200 mg group) experienced constipation, compared with four patients in the placebo group. In contrast, GI AEs commonly associated with sevelamer (reported in an average of 38% of patients) were predominantly constipation, nausea and upper abdominal pain/discomfort [6, 8]. These GI AEs should be considered in the context of end-stage renal disease, where more than half of patients on dialysis experience constipation [31].

Iron accumulation is a potential concern in dialysis patients. In this study there were no major shifts in iron measures and PT20 use over 28 days had no effect on haemoglobin or ferritin concentrations or transferrin saturation. A more pronounced effect on iron measures has been reported with ferric citrate, whereas sucroferric oxyhydroxide showed smaller changes in iron measures with no indication of iron accumulation [14, 32, 33]. The differences in effects on iron measures among these agents may be attributable to differences in the compounds: both PT20 and sucroferric oxyhydroxide have been designed to minimize iron release and absorption [34].

The pharmacokinetics of adipate administered in supraphysiological quantities to haemodialysis patients are unknown. Adipic acid is probably metabolized by β-oxidation in a similar manner to fatty acids, but these processes may be altered in end-stage renal disease [35, 36]. For example, it has been shown that serum adipate is elevated in dialysis-dependent patients but levels can be decreased significantly by haemodialysis [35, 36]. Increased adipate exposure from PT20 is a potential concern among dialysis-dependent patients, in whom serum adipate is already elevated and acidosis is prevalent. Importantly, we found that treatment with PT20 caused no major shifts in chemistry reflective of changes in acid/base status.

Limitations of this Phase 2 study include the relatively small sample size and short study duration of 1 month. The fixed-dose study design also limits assessment of the real-world potential of PT20 for the treatment of patients with hyperphosphataemia. Patients with lung disease or high serum ferritin levels (≥1000 ng/mL) were excluded from the study population, so the findings cannot be generalized to these patient groups.

In conclusion, PT20 treatment lowered serum phosphate concentration in patients with dialysis-dependent CKD and hyperphosphataemia. PT20 was generally well tolerated across a wide dosing range, suggesting that it could be titrated to address hyperphosphataemia in patients with varying levels of disease and dietary phosphate. These results indicate that PT20 shows promise as a phosphate binder and further evaluation of its efficacy and safety in larger clinical trials is warranted.
